# Enhanced facial grimacing when laparotomy involves cutaneous and visceral tissue injury

**DOI:** 10.1097/PR9.0000000000001275

**Published:** 2025-04-28

**Authors:** Minghao Shao, Eric S. McCoy, Mark J. Zylka

**Affiliations:** a UNC Neuroscience Center, The University of North Carolina at Chapel Hill, Chapel Hill, NC, USA; b Department of Cell Biology & Physiology, The University of North Carolina at Chapel Hill, Chapel Hill, NC, USA

**Keywords:** Grimace, Laparotomy, CD-1, C57Bl/6, Pain, Visceral

## Abstract

**Introduction:**

Laparotomy is a common surgical procedure that entails incision of the abdomen and is associated with varying degrees of postoperative pain. Laparotomies can be performed in mice and result in facial grimacing, which can be quantified using PainFace, a software platform that automates facial grimace analyses.

**Objectives:**

We evaluated the extent to which incision of the ventral skin, peritoneum, and intestinal manipulation, all of which can occur as part of a laparotomy surgery, affects the magnitude and duration of facial grimacing in 2 strains of mice along with allodynia at the incision site in CD-1 mice.

**Methods:**

White-coated CD-1 male and female mice and black-coated C57BL/6 male mice (8-12 weeks of age) were split into groups (n = 20 per group) that underwent laparotomies with varying manipulations.

**Results:**

Mouse grimace scale scores were higher in both strains after surgery when the small intestine was manipulated in 2 different ways compared to groups that received a cutaneous incision alone or cutaneous and peritoneal incision.

**Conclusion:**

These studies show that mice exhibit more pronounced facial grimacing when both cutaneous and visceral tissues are injured during laparotomy surgery. Consistent with clinical findings, our experiments suggest that postoperative pain could be reduced by minimizing visceral tissue injury during surgical procedures.

## Abstract 2

Supplemental Digital Content is Available in the Text.

Facial grimacing in mice is more pronounced when cutaneous and visceral tissues are injured during laparotomy surgery when compared to cutaneous injury alone.

## 1. Introduction

Postoperative pain is intrinsic to surgery and can lead to chronic postsurgical pain (CPSP) in approximately 10% of all surgical patients.^12^ Certain procedures, like limb amputation, have up to 85% CPSP prevalence.^10^ With an annual 313 million surgical operations being conducted globally,^16^ the impact of CPSP demands treatment with the support of translational research. Many animal models have been developed to study postsurgical pain in rodents,^1,4,6,19^ including laparotomy models that feature spontaneous pain.^17,22^

The mouse grimace scale (MGS) was developed to quantify facial expressions indicative of pain.^11^ Scoring was traditionally done by humans, making the scoring process laborious and time-consuming.^11,23,26^ To automate scoring, we recently developed PainFace, a software platform that employs machine learning to accurately predict MGS scores. PainFace can quantify facial grimacing and analgesic-mediated relief of facial grimacing after laparotomy surgery in black-coated C57BL/6 mice^15^ and white-coated mice with accuracies comparable to highly trained human scorers.

Prior studies utilized laparotomy models like peritoneal abrasion,^14^ incisions modeling vasectomies or embryonic manipulations,^7^ and colon abrasions.^13^ In the rat plantar incision model of postoperative pain, injury of the skin and muscle resulted in more mechanical hyperalgesia relative to injury of the skin alone.^5^ To our knowledge, no manipulation-specific characterization of laparotomy has been performed. Here, we sought to evaluate the extent to which cutaneous incision alone, epithelium and peritoneal incision, or epithelium incision, peritoneal incision, and visceral manipulation influenced the magnitude and duration of facial grimacing and allodynia in mice.

## 2. Methods

### 2.1. Animal care and use

All procedures used in this study were approved by the Institutional Animal Care and Use Committee at the University of North Carolina at Chapel Hill. Sex-separated male and female group–housed CD-1 mice (8-12 weeks of age; Charles Rivers Laboratories, Wilmington, MA) and male group–housed C57BL/6 mice (8-12 weeks of age; The Jackson Lab, Bar Harbor, ME) were maintained on a 12:12 hour light/dark cycle and given food (PicoLab 5V5R; LabDiet, Richmond, IN) and tap water ad libitum.

### 2.2. Laparotomy surgeries

**Figure 1.**
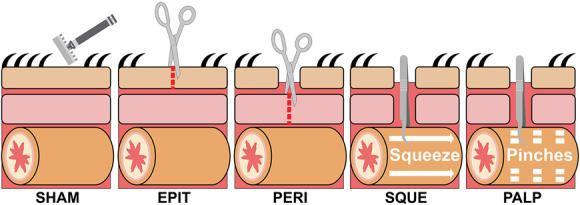
Laparotomy surgery manipulations. SHAM, Fur shaved with a razor; EPIT, epithelium incision; PERI, peritoneum incision; SQUE, small intestine gently squeezed; PALP, small intestine palpated.

The mouse laparotomy procedure involves abdominal shaving followed by epithelium and peritoneum incisions, and intestinal manipulation.^15^ To evaluate how components of this surgical procedure influence facial grimacing, we separated mice into experimental groups. SHAM mice received only the abdominal shave, EPIT mice received the shave and epithelial incision, and PERI mice received the shave and both epithelial and peritoneum incisions (Fig. 1). The SQUE mice were shaved, received both epithelial and peritoneum incisions, and had approximately 1 to 2 cm of intestines exteriorized, squeezed with forceps, and pulled downward 1 to 2 cm (Fig. 1). For the PALP mice, approximately 1 to 2 cm of intestines were exteriorized and gently pinched 10 times (Fig. 1). Postsurgery, mice recovered on a heated pad for 30 minutes. Mice were returned to the imaging chambers and recorded for 30 minutes. Subsequent 30-minute recordings were taken 2 hours and 4 hours postsurgery.

### 2.3. von Frey experiments

Female CD-1 mice were acclimated to the von Frey station for 3 days. Each mouse was probed for 3 seconds with a 0.4-g von Frey filament near the prospective laparotomy incision site. Five stimulations were conducted each acclimation day. On the experimental day, the baseline sensitivity score was recorded as described.^24^ For each of the 5 stimulations, a score of “0” was measured with no response, a “1” for licking or movement of the abdomen, a “2” for flinching, and a “3” for the mouse jumping or moving around the chamber. The 5 scores were summed to yield the sensitivity score. Mice with baseline sensitivity scores greater than 6 were removed from the study, as was done by Schiene et al.^20^ Each mouse then underwent surgery. Three hours after surgery, another sensitivity score was collected per mouse.

### 2.4. Data analysis

All videos were recorded at 30 frames per second (fps) for 30 minutes and analyzed by PainFace for facial grimaces at 1 fps. For white-coated CD-1 mice build 20231114-white-amgsnet was used. For black-coated C57BL/6 mice build 20221115-black was used. Only frames containing the maximum number of facial action units (MaxFAU: 5 FAUs for white-coated mice—orbitals, ears, whiskers, nose, and cheeks; 4 FAUs for black-coated mice—orbitals, ears, whiskers, and nose) were used. Videos included in the analysis met a threshold of MaxFAU frames equal to or greater than 12.5% of total video frames, equivalent to 225 MaxFAU frames for the 30-minute videos. Videos short of this threshold were excluded from analysis.

Statistical analyses were performed in GraphPad Prism 9. Statistical significance values were obtained with Kruskal–Wallis test performed with Dunn post-hoc test. Male and female CD-1 MGS score comparisons used the Mann–Whitney *U* test.

## 3. Results

**Figure 2.**
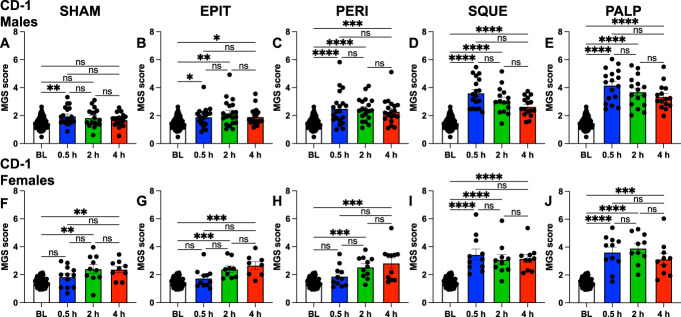
Male (A–E) and female (F–J) CD-1 laparotomy surgical manipulation groups. PainFace used to analyze facial grimaces from each 30-min video. Mean mouse grimace scale (MGS) scores from (A and F) SHAM, (B and G) EPIT, (C and H) PERI, (D, I) SQUE, and (E and J) PALP groups at baseline (BL), 0.5-hour, 2-hour, and 4-hour time points. Error bars represent standard error of the mean (sem). Kruskal–Wallis test performed with Dunn post-hoc test. *P < 0.05, **P < 0.01, ***P < 0.001, ****P < 0.0001. Male, n = 15 to 20 per group. Female, n = 8 to 12 per group.

Male and female CD-1 mice were examined at 0.5 hour, 2 hours, and 4 hours after laparotomy for facial grimacing (Fig. 2). A significant mean MGS score increase was seen at some or all time points for each of the postsurgery groups relative to the combined baseline (Figs. 2A–J). PALP showed the highest mean MGS score increases, followed by SQUE (Figs. 2D–E, I–J). Comparison of male and female CD-1 mice MGS scores at the 0.5-hour time point for the same surgical groups showed no significant differences (Supplementary Fig. 1, http://links.lww.com/PR9/A304).

**Figure 3.**
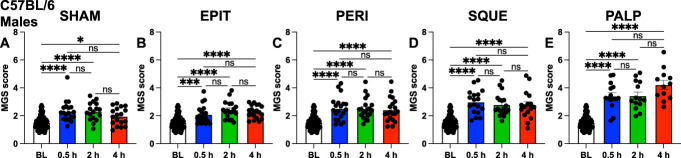
Male C57BL/6 laparotomy surgical manipulation groups. PainFace used to analyze facial grimaces from each 30-min video. Mean mouse grimace scale (MGS) scores from (A) SHAM, (B) EPIT, (C) PERI, (D) SQUE, and (E) PALP groups at baseline (BL), 0.5-hour, 2-hours, and 4-hour time points. Error bars represent sem. Kruskal–Wallis test performed with Dunn post-hoc test. *P < 0.05, ***P < 0.001, ****P < 0.0001. n = 12 to 20 per group.

For male C57BL/6 mice, all groups displayed a significant increase in mean MGS scores at all postsurgery time points compared to the combined baseline (Figs. 3A–E). PALP had the highest mean MGS score increases (Fig. 3E), followed by SQUE (Fig. 3D), relative to the remaining groups (Figs. 3A–C). Baseline values were similar for all surgical groups before surgery with CD-1 and C57BL/6 mice. For all MGS score analyses with C57BL/6 and CD-1 mice, only minimal differences in mean MaxFAU frames were found (Supplementary Fig. 2, http://links.lww.com/PR9/A304).

**Figure 4.**
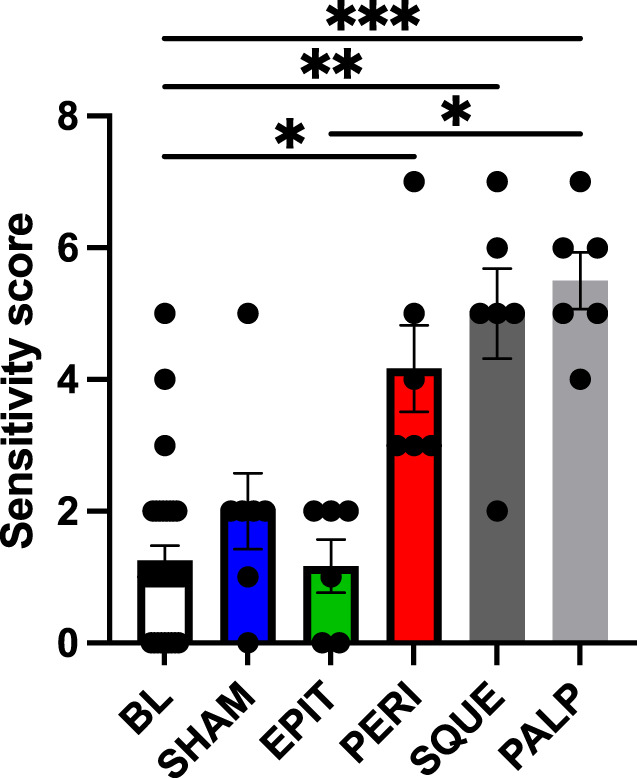
Mechanical allodynia at incision site in female CD-1 mice. Mean sensitivity scores for SHAM, EPIT, PERI, SQUE, and PALP groups measured 3 hours after laparotomy relative to baseline (BL, combined values from all groups). Error bars represent sem. Kruskal–Wallis test performed with Dunn post-hoc test. *P < 0.05, **P < 0.01, ***P < 0.001. n = 6 to 7 per group.

We also measured allodynia at the incision site 3 hours after laparotomy in the same female CD-1 mice used for the grimace analyses above. The mechanical sensitivity scores for PERI, SQUE, and PALP groups were significantly higher compared to the combined baseline group (Fig. 4). The SQUE and PALP groups showed the highest sensitivity score increases, consistent with higher mean MGS scores in these groups relative to the other groups.

## 4. Discussion

We found that mean MGS scores were higher in surgical groups where visceral tissues were injured (SQUE and PALP) compared to groups where only superficial tissues were shaved or injured (SHAM, EPIT, PERI) in male and female CD-1 mice and in male C57BL/6 mice. These data suggest that SQUE and PALP surgical manipulations, which presumably generate visceral nociceptive pain, resulted in greater mouse facial grimacing in inbred and outbred mice when compared to animals that received more superficial injury alone. The SQUE and PALP groups also showed the greatest increase in allodynia at the incision site, as assessed via the sensitivity score. Rats likewise displayed greater mechanical hyperalgesia and decreased withdrawal thresholds when the plantaris muscle and the overlying skin was incised when compared to incision of the plantar skin alone.^5^ In the Brennan rat plantar model and our study using the mouse laparotomy model, rodents undergoing sham procedures displayed a modest increase in allodynia and facial grimace, respectively, suggesting that nonincisional procedures cause nominal increases in allodynia/grimace responses.

The selection of mouse strain is important when evaluating any surgical model. Previous research found that inbred mouse strains differ in reaction to surgical procedures and nociceptive pain responses.^21^ The MGS score range is greater in white-coated mice (0-10) with 5 facial action units relative to black-coated mice (0-8) with 4 facial action units.^11,15^ This broader scoring scale for CD-1 mice explains the slightly higher mean MGS scores postlaparotomy compared to C57BL/6 mice. Moreover, we found that mean MGS scores were similar between male and female CD-1 mice before and after laparotomy.

We speculate that mice grimace to a greater extent when laparotomy affects the viscera because of convergence between visceral and cutaneous nociceptive afferents within the central nervous system. The small intestine is richly innervated by afferent fibers from enteric, vagal, and spinal neurons.^3^ Moreover, visceral injury can cause referred pain^9^ due to the convergence of primary sensory neurons at second-order neurons.^2,8^ In cases of human abdominal trauma, laparoscopy causes less disturbance to visceral tissues when compared to laparotomy surgery, and shorter hospitalization time is associated with this less invasive procedure.^25^ To limit visceral injuries during laparoscopies, reduced-port techniques further reduce injury and invasiveness.^18^

Overall, our study highlights the importance of producing some level of visceral tissue injury to model postoperative pain. The approaches in our study could be expanded to other postoperative pain models and reinforce clinical observations that less-invasive laparoscopic techniques can potentially reduce recovery time and postoperative pain.^25^

## Disclosures

The authors have no conflicts of interest to declare.

## Appendix A. Supplemental digital content

Supplemental digital content associated with this article can be found online at http://links.lww.com/PR9/A304.

## Supplementary Material

SUPPLEMENTARY MATERIAL
