# Supplementary material for: Enhanced facial grimacing when laparotomy involves cutaneous and visceral tissue injury
Source: Pain Rep. 2025 Apr 28;10(3):e1275. doi: 10.1097/PR9.0000000000001275 (PMC12039984; doi:10.1097/PR9.0000000000001275)
Supplement: SUPPLEMENTARY MATERIAL [file painreports-10-e1275-s001.pdf]

Supplementary Data

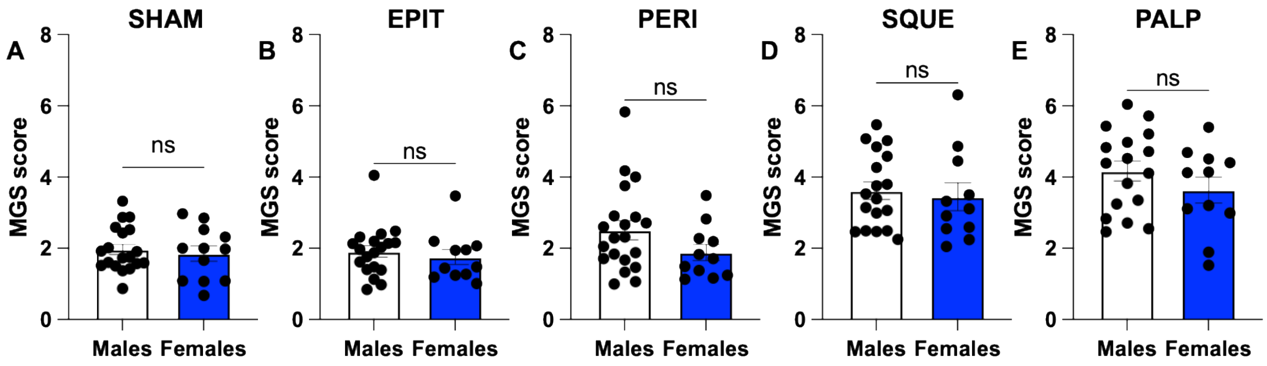

Supplementary Figure 1. Comparison of male and female CD-1 laparotomy surgical manipulation groups, (A) SHAM, (B) EPIT, (C) PERI), (D) SQUE, and (E) PALP, for differences in mean MGS score at 0.5 h timepoint. Error bars represent sem. Mann-Whitney U test performed. n = 11-20/group.

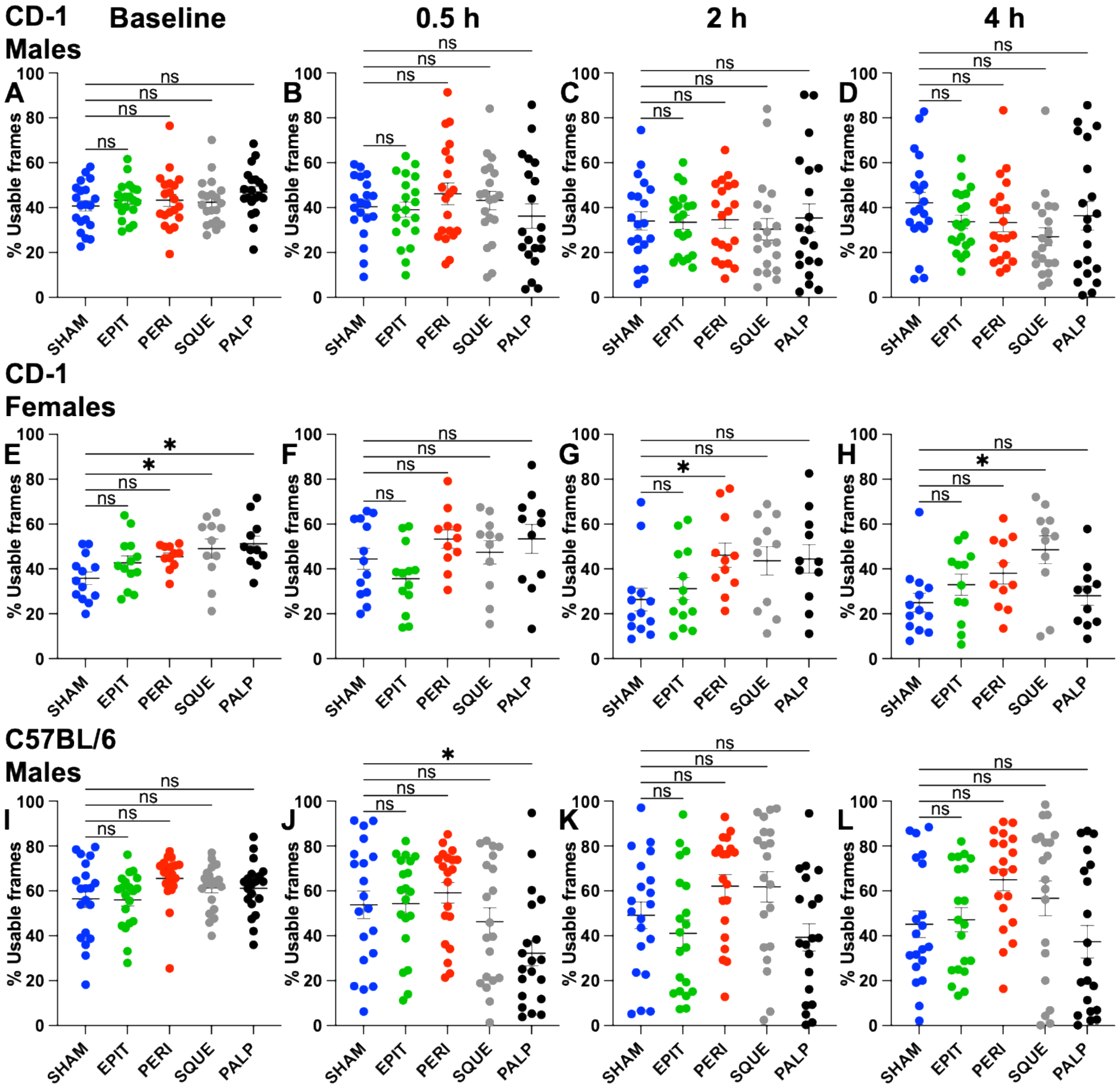

Supplementary Figure 2. Percentage of usable frames (MaxFAU). Five FAUs for male (A-D) and female (E-H) CD-1 mice. Four FAUs for male C57BL/6 mice (I-L). (A,E,I) Baseline, (B,F,J) 0.5 h, (C,G,K) 2 h, and (D,H,L) 4 h timepoints for all surgical groups. Error bars represent sem. Kruskal-Wallis test performed with Dunn’s post-hoc test. *p < 0.05. n = 11-20/group.
